# Comparison of statins for secondary prevention in patients with ischemic stroke or transient ischemic attack: a systematic review and network meta-analysis

**DOI:** 10.1186/s12916-019-1298-5

**Published:** 2019-03-26

**Authors:** Irene Tramacere, Giorgio B. Boncoraglio, Rita Banzi, Cinzia Del Giovane, Koren H. Kwag, Alessandro Squizzato, Lorenzo Moja

**Affiliations:** 1 Department of Research and Clinical Development, Fondazione IRCCS Istituto Neurologico Carlo Besta, 20133 Milan, Italy; 2 Department of Cerebrovascular Disease, Fondazione IRCCS Istituto Neurologico Carlo Besta, Milan, Italy; 3 Center for Drug Regulatory Policies, Istituto di Ricerche Farmacologiche Mario Negri IRCCS, Milan, Italy; 4 Institute of Primary Health Care (BIHAM), University of Bern, Bern, Switzerland; 5 Medical School for International Health, Ben Gurion University, Beersheba, Israel; 6 Department of Medicine and Surgery, University of Insubria, Varese, Italy; 7 Department of Biomedical Sciences for Health, University of Milan, Milan, Italy; 8 Unit of Clinical Epidemiology, IRCCS Galeazzi Orthopedic Institute, Milan, Italy

**Keywords:** Systematic review, Network meta-analysis, RCT, Stroke, Secondary prevention, Statins

## Abstract

**Background:**

Statins may prevent recurrent ischemic events after ischemic stroke. Determining which statin to use remains controversial. We aimed to summarize the evidence for the use of statins in secondary prevention for patients with ischemic stroke by comparing benefits and harms of various statins.

**Methods:**

We searched for randomized controlled trials (RCTs) assessing statins in patients with ischemic stroke or transient ischemic attack (TIA) in MEDLINE, EMBASE, and CENTRAL up to July 2017. Two authors extracted data and appraised risks of bias. We performed pairwise meta-analyses and trial sequential analyses (TSA) to compare statins versus placebo/no statin, and network meta-analyses using frequentist random-effects models to compare statins through indirect evidence. We used GRADE to rate the overall certainty of evidence. Primary outcomes were all-cause mortality and all strokes. Secondary outcomes were different types of strokes, cardiovascular events, and adverse events.

**Results:**

We identified nine trials (10,741 patients). No head-to-head RCTs were found. The median follow-up period was 2.5 years. Statins did not seem to modify all stroke and all-cause mortality outcomes; they were associated with a decreased risk of ischemic stroke (odds ratio, OR, 0.81 [95% CI, 0.70 to 0.93]; absolute risk difference, ARD, − 1.6% [95% CI, − 2.6 to − 0.6%]), ischemic stroke or TIA (OR, 0.75 [95% CI, 0.64 to 0.87]; ARD, − 4.2% [95% CI, − 6.2 to − 2.1%]), and cardiovascular event (OR, 0.75 [95% CI, 0.69 to 0.83]; ARD, − 5.4% [95% CI, − 6.8 to − 3.6%]), and did not seem to modify rhabdomyolysis, myalgia, or rise in creatine kinase. In the comparison of different statins, moderate- to high-quality evidence indicated that differences between pharmaceutical products seemed modest, with high doses (e.g., atorvastatin 80 mg/day and simvastatin 40 mg/day) associated with the greatest benefits. TSA excluded random error as a cause of the findings for ischemic stroke and cardiovascular event outcomes. Evidence for increased risk of hemorrhagic stroke was sensitive to the exclusion of the SPARCL trial.

**Conclusions:**

Evidence strongly suggests that statins are associated with a reduction in the absolute risk of ischemic strokes and cardiovascular events. Differences in effects among statins were modest, signaling potential therapeutic equivalence.

**Trial registration:**

PROSPERO CRD42018079112

**Electronic supplementary material:**

The online version of this article (10.1186/s12916-019-1298-5) contains supplementary material, which is available to authorized users.

## Background

Stroke is the second most common cause of death in industrialized countries and the leading cause of permanent acquired disability [1, 2]. Although a transient ischemic attack (TIA) does not leave any impairment, affected individuals are at a high risk for future ischemic events, particularly in the days and weeks immediately following symptom resolution [3]. On average, the annual risk of future ischemic stroke after an initial stroke or TIA is 3 to 5% [4]. Patients with ischemic stroke or TIA are also at a higher risk for subsequent myocardial infarction and death from vascular causes [4–6].

Since the risk of ischemic stroke is higher in the early period after the acute event, prompt initiation of tailored prevention strategies is essential [7]. It has been estimated that at least 80% of recurrent ischemic events in patients with previous ischemic stroke may be prevented through the use of a comprehensive approach that includes dietary modification, exercise, antiplatelet/anticoagulant therapy, effective strategies for treatment of hypertension, and statins [8].

Historically, the role of statin therapy in the prevention of cardiovascular disease has been well received by the medical and scientific communities, with numerous guidelines promoting their use. Specifically, the 2013 ACC/AHA guideline recommends the use of statins to reduce the risk of stroke and cardiovascular events in patients with ischemic stroke or TIA presumed to be of atherosclerotic origin [6]. Possible adverse events that have been associated with statin therapy are myopathy and hemorrhagic stroke [5, 6, 9].

Despite the widespread use of statins, however, the relative safety and efficacy of different statin drugs have not been clearly defined. In fact, no head-to-head trial has directly addressed the effectiveness of these therapies or has clearly defined the choice of a specific statin, dose (low, moderate, or high), and target (all ischemic stroke and TIA or non-cardioembolic only or high LDL cholesterol only) [5, 6, 9].

A more accurate understanding of the effectiveness and safety of different statins is required to make appropriate drug choices, both at an individual and public health level, informing drug prescribing and procurement. In this network meta-analysis, we aim to summarize the current evidence for statin use in secondary prevention of patients with ischemic stroke or TIA by estimating the relative efficacy and safety of various statins and providing a final ranking of the different molecules. Our results may inform clinicians in their daily practice as well as scientific societies and agencies developing best practice guidelines.

## Methods

### Protocol and registration

The systematic review protocol was developed using guidance from the Preferred Reporting Items for Systematic Review and Meta-analysis Protocols (PRISMA-P) statement [10]. We addressed all 17 items within the PRISMA-P checklist and registered the review in PROSPERO (CRD42018079112) [11]. We used the PRISMA-network meta-analysis extension to report the results [12].

### Search strategy and selection criteria

We conducted a systematic review and network meta-analysis. We searched MEDLINE, EMBASE, and the Cochrane Central Register of Controlled Trials (CENTRAL) electronic databases from January 2008 to July 2017, with no language restrictions. Search terms included extensive controlled vocabulary (MeSH and EMTREE) and keywords, including the names of statins along with differing terms for stroke and cerebrovascular disease in various combinations. Details on the search strategies can be found on PROSPERO (https://www.crd.york.ac.uk/prospero/display_record.php?RecordID=79112). Studies published before January 2008 were retrieved from two Cochrane reviews featuring similar PICOs and a broader inclusion criteria of studies [13, 14]. We did not formally search for additional unpublished or ongoing studies as we did not identify additional studies relevant to our review question during a preliminary check on ClinicalTrials.gov (http://www.clinicaltrial.gov/) and the International Clinical Trials Registry Platform (ICTRP) (http://apps.who.int/trialsearch/).

### Eligibility criteria

We included randomized controlled trials (RCTs) comparing any single statin at any dose with either a control (placebo/no statin) or another active statin for secondary prevention in adults (≥18 years old, both sexes) diagnosed with ischemic stroke or TIA in which hemorrhage had been excluded by neuroimaging. We included all settings of care (e.g., acute or nursing homes, hospitals or ambulatory, primary or secondary, inpatients or outpatients), as well as both acute and delayed treatments. RCTs comparing the effect of different doses of the same statin were excluded, except those that included another eligible comparator. We assumed that medications were “jointly randomizable” across patients included in the trials. In other words, a patient could have been, in principle, randomized to any of the alternative treatment options; it is, therefore, possible to imagine that a single randomized trial could have been designed to compare all of these treatments (transitivity assumption) [15]. We excluded non-English-language study reports.

Two authors independently selected the studies, reviewed the main reports and supplementary materials, extracted relevant information from the included trials, and assessed the risk of bias. Any discrepancies were resolved by consensus and arbitration by a third author.

### Outcomes

Primary outcomes were all-cause mortality and the proportion of patients who developed a stroke after statin use, irrespective of its type (ischemic or hemorrhagic) and severity. Secondary outcomes included the proportion of patients who developed an ischemic stroke in which hemorrhage had been excluded by imaging or autopsy; an ischemic stroke or TIA irrespective of severity; a hemorrhagic stroke, defined as an acute extravasation of blood into the brain parenchyma that excludes subarachnoid hemorrhage, subdural hematoma, and epidural hematoma [16]; a cardiovascular event defined as any sudden death, fatal or non-fatal acute coronary syndrome, stroke, intracranial hemorrhage, or pulmonary embolism; and rhabdomyolysis, myalgia, or rise in creatine kinase (CK). For all analyses, we recorded the outcomes at the longest available follow-up.

We used network plots to describe the network geometry [17].

### Risk of bias assessment and certainty of evidence

We evaluated the risk of bias for each included study using the criteria of The Cochrane Collaboration [18]. The following domains of bias were considered: selection (random sequence generation, allocation concealment), performance, detection (blinding of participants and personnel, and of outcome assessment), attrition (incomplete outcome data), selective outcome reporting, and the role of the sponsor in the authorship of the study report or in data management or analysis. We explicitly judged the risk of bias in each criterion as “low,” “high,” or “unclear.” We considered the blinding of participants, personnel, and outcome assessment separately for objective outcomes (e.g., mortality) and subjective outcomes (myalgia). We evaluated incomplete outcome data as having a low risk of bias when the numbers and reasons for dropouts were balanced (i.e., in the absence of a significant difference) between arms. Our assessment of methodological quality included published trial protocols, when available. To summarize the quality of the evidence, we considered allocation concealment, blinding of outcome assessment, and incomplete outcome data; we classified each study as having a low risk of bias when all three criteria were evaluated at a low risk of bias; a high risk of bias when at least one criterion was at high risk of bias; and a moderate risk of bias in the remaining cases. This appraisal was conducted by pairs of independent reviewers, with conflicts resolved by a third reviewer. We examined the overall certainty of the evidence for primary and secondary outcomes using the Grading of Recommendation, Assessment, Development and Evaluation (GRADE) framework methodology [19]. We revised and assessed each GRADE item (study limitations, inconsistency, indirectness, imprecision, and publication bias) considering issues related specifically to the network meta-analysis methodology: the presence of indirect comparisons, the influence of each direct piece to the network meta-analysis evidence, and the role of the consistency assumption for the validity of the estimates [20].

### Statistical analyses

We estimated treatment effects from each study using the odds ratio (OR) with 95% confidence intervals (95% CIs). For all outcomes with at least two studies, we performed standard pairwise meta-analyses of any statin versus placebo/no statin with a random-effects model. We compared different statins through network meta-analyses performed under a frequentist framework using a random-effects model. Results of network meta-analyses were presented in league tables and forest plots. For each outcome, we estimated the probability of each treatment included in the network to be the best among all treatments by using the surface under the cumulative ranking curve area (SUCRA) [21]. We presented the results from pairwise meta-analyses and the network meta-analysis as summary relative effect sizes. We reported absolute risk difference (ARD) estimates, calculated using as baseline the proportion of patients with an event in the control arm of the included studies, and applying the OR estimated in the meta-analysis to compute the absolute difference between the intervention and control arms. In the standard pairwise meta-analyses, we assessed clinical heterogeneity by comparing the data on potential effect modifiers, and determined the presence of statistical heterogeneity by visual inspection of the forest plots and calculation of the *I*^2^ statistic [22]. We assumed a common estimate for the heterogeneity variance across treatment comparisons in the network meta-analysis. We compared the distribution of potential effect modifiers across different pairwise comparisons to assess transitivity across treatment comparisons. We checked for the presence of statistical heterogeneity in the entire network by considering the magnitude of the common heterogeneity parameter [23]. As only direct evidence was available, we were not able to assess incoherence (defined as the statistical disagreement between direct and indirect evidence) within each network. We performed subgroup analyses of any statin versus placebo/no statin considering the following potential sources of heterogeneity (effect modifiers): stroke subtypes at inclusion, treatment dose intensity as defined in the 2013 ACC/AHA Guideline [24], and time from the first ischemic event to randomization. We performed sensitivity analyses of any statin versus placebo/no statin for each primary and secondary outcome, including only trials that were classified as having a low risk of bias. In order to control for the risks of type I and type II errors due to sparse data and repetitive testing of accumulating data, a trial sequential analysis of any statin versus placebo/no statin was performed for each primary and secondary outcome with at least two studies. Each trial sequential analysis provided the required information size for a meta-analysis and calculated the adjusted statistical thresholds for benefits, harms, or futility before the required information size was reached. The following assumptions were used: the proportion of participants in the control group with events; a relative risk reduction or an increase of 10% for primary outcomes, and of both 10% and 20% for secondary outcomes; a type I error of 5%; a type II error of 20%; and the observed diversity of the meta-analysis [25–28].

Finally, we performed a post hoc sensitivity analysis, which was prompted by external reviewer comments: we used a leave-one-out meta-analysis to assess the independent influence of each study on the summary estimate [29].

## Results

**Fig. 1:**
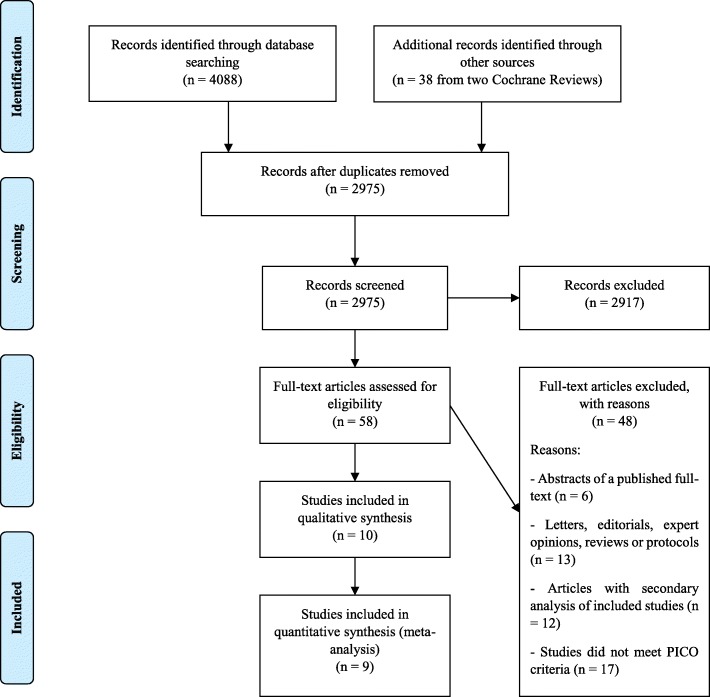
Study selection

A total of 2975 citations were identified by the search, and 58 potentially eligible articles were retrieved in full-text (Fig. 1). Overall, ten trials were included in the review [30–39]. With the exception of Plehn 1999 [30], which did not report separate results for patients with ischemic stroke at inclusion, the remaining nine studies presented data suitable for meta-analysis (*n* = 10,741 patients). Table 1 summarizes the characteristics of included studies. In three trials, the lipid-lowering drug was compared to no statin [35–37], while the remaining trials compared the drug to placebo [30–34, 38, 39]. Atorvastatin was studied in a single large trial [33], while simvastatin was studied in four trials [32, 34, 35, 39], pravastatin in three [30, 31, 36], and rosuvastatin in two [37, 38]. Across studies, most patients were randomized to atorvastatin (*n* = 2365), followed by simvastatin (*n* = 1870), pravastatin (*n* = 1060), and rosuvastatin (*n* = 167). Based on dose intensity defined in the 2013 ACC/AHA Guideline [24], one out of ten trials investigated statins at a low-intensity dose (i.e., pravastatin 10 mg/day) [36], seven at a moderate-intensity dose (i.e., pravastatin 40 mg/day, rosuvastatin 5 mg/day, or simvastatin 40 mg/day) [30–32, 34, 35, 37, 39], and two at a high-intensity dose (i.e., atorvastatin 80 mg/day or rosuvastatin 20 mg/day) [33, 38]. Among the eight RCTs reporting information on the time from the first ischemic event to randomization, five studies randomized patients within 1 week of the first event [34, 35, 37–39], and three studies at least 1 month after the first event [32, 33, 36]. Six of ten RCTs randomized patients with both cardioembolic and non-cardioembolic ischemic stroke [30–32, 34, 35, 39], and four RCTs randomized only patients with a non-cardioembolic ischemic stroke at inclusion [33, 36–38]. When the information was available, patients were more frequently men (54–96%), with the exception of two RCTs in which men were 44% and 48% of the study population [34, 35]. The mean range of age was 63–74 years. All five RCTs that had available information randomized patients with mild to moderate severity stroke at inclusion (mean National Institutes of Health Stroke Scale, NIHSS, at baseline lower than 15) [34, 35, 37–39]. The median follow-up period was 2.5 years (range, 5 days–6 years), and four RCTs [31–33, 36] had a follow-up period of at least 4 years.

**Table 1:** Characteristics of the included studies

Study	Intervention	Control	Country	Recruitment period	Time from first ischemic event to randomization	Stroke subtype at inclusion^a^	Age (mean)	Sex (% males)	Follow-up
Plehn 1999 [30]	Pravastatin 40 mg/day	Placebo	USA and Canada	Dec 1989–Dec 1991	NA	Both cardio and non-cardio	NA	NA	5 years
White 2000 [31]	Pravastatin 40 mg/day	Placebo	Australia and New Zealand	June 1990–Dec 1992	NA	Both cardio and non-cardio	NA	NA	6 years
Collins 2004 [32]	Simvastatin 40 mg/day	Placebo	UK	July 1994–May 1997	> 6 months	Both cardio and non-cardio	66	75%	5 years
Amarenco 2006 [33]	Atorvastatin 80 mg/day	Placebo	World	Sept 1998–Mar 2001	1–6 months	Non-cardio	63	60%	4 years
Kennedy 2007 [34]	Simvastatin 40 mg/day	Placebo	USA and Canada	May 2003–Dec 2006	≤ 24 h	Both cardio and non-cardio	68	48%	90 days
Yakusevich 2012 [35]	Simvastatin 40 mg/day	No statin	Russia	2008–2010	24–48 h	Both cardio and non-cardio	66	44%	1 year
Hosomi 2015 [36]	Pravastatin 10 mg/day	No statin	Japan	Mar 2004–Feb 2009	1 months–3 years	Non-cardio	66	69%	5 years
Ueno 2015 [37]	Rosuvastatin 5 mg/day	No statin	Japan	Aug 2011–Sept 2013	≤ 7 days	Non-cardio	71	96%	6 months
Heo 2016 [38]	Rosuvastatin 20 mg/day	Placebo	Korea	Aug 2010–June 2013	≤ 66 h	Non-cardio	65	60%	5 or 14 days
Montaner 2016 [39]	Simvastatin 40 mg/day	Placebo	Spain	Apr 2009–Mar 2014	≤ 12 h	Both cardio and non-cardio	74	54%	90 days

^a^*Cardio* cardioembolic ischemic stroke, *Non-cardio* non-cardioembolic ischemic stroke

**Fig. 2:**
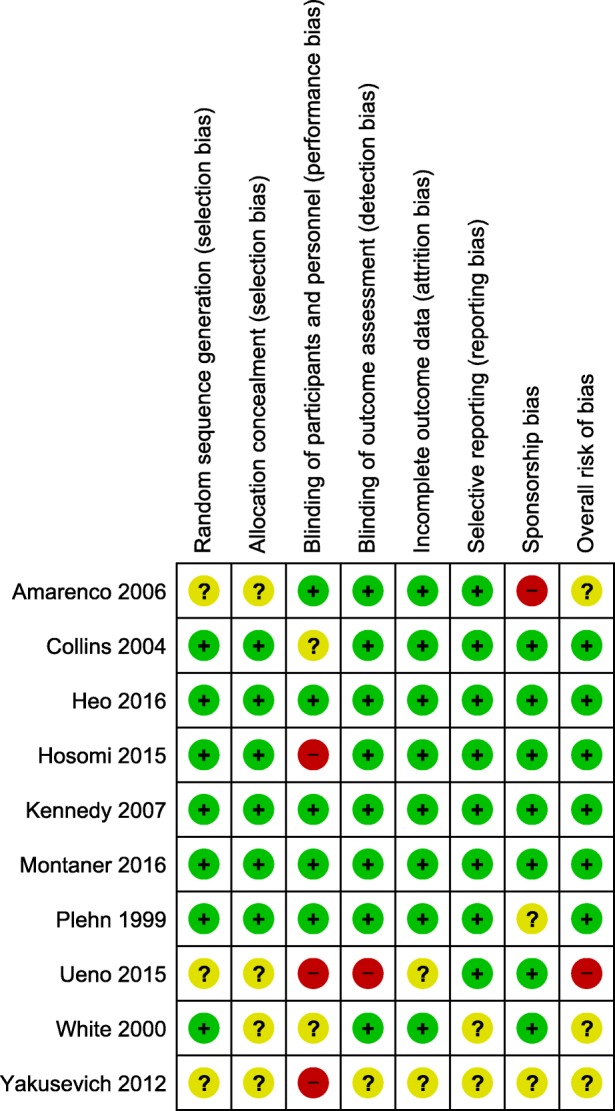
Risk of bias of the included studies

Overall, one (10%) trial was rated as high risk of bias, three (30%) trials as moderate, and six (60%) trials as low (Fig. 2). By using GRADE, we rated the quality of evidence as high for ischemic stroke, ischemic stroke or TIA, and cardiovascular event; moderate for all strokes; and low for all-cause mortality, hemorrhagic stroke, and rhabdomyolysis, myalgia, or rise in CK outcomes.

**Fig. 3:**
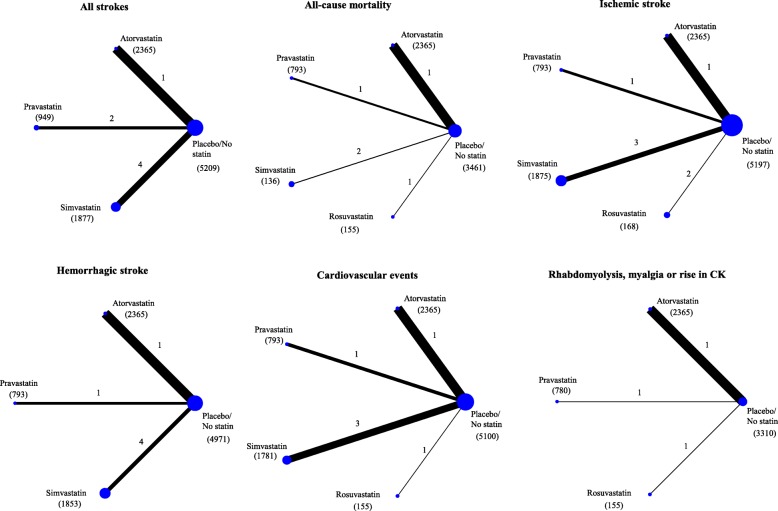
Network plots of evidence for primary and secondary outcomes: each line links the treatments that have been directly compared in studies. The thickness of the line is proportional to the precision of each direct estimate, and the width of each circle is proportional to the number of studies included in the treatment. The number of studies per comparison is reported next to each line, and the number of patients included in each treatment is reported in the bracket below the treatment name

**Fig. 4:**
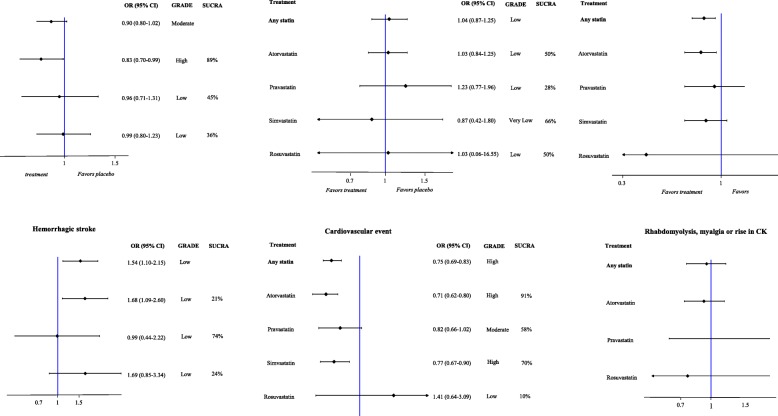
Forest plot of meta-analysis estimates of any statin against placebo/no statin, and of each treatment against placebo/no statin within the networks for primary and secondary outcomes with at least two studies, with the corresponding ranking probability (SUCRA) and quality of evidence (GRADE) for each intervention for each outcome

Figure 3 shows the network geometry for each primary and secondary outcome. Figure 4 shows the estimates of primary and secondary outcomes of any statin against placebo/no statin from the standard meta-analysis, and of each statin against placebo/no statin from the network meta-analysis, with the corresponding ranking probability (SUCRA) and quality of evidence (GRADE) for each outcome. Additional file 1: Tables S1-S6 shows the network meta-analysis estimates of primary and secondary outcomes for each comparison between different statins and placebo/no statin.

Seven RCTs, comprising 10,398 patients, addressed all stroke outcomes comparing a statin versus placebo/no statin. Moderate-quality evidence indicated that 10.4% patients taking a statin experienced a stroke (ischemic or hemorrhagic) compared with 11.3% patients taking placebo/no statin (OR, 0.90 [95% CI, 0.80 to 1.02]; ARD, − 1.0% [95% CI, − 2.1 to 0.2%]). By comparing different statins, high-quality evidence indicated that atorvastatin 80 mg/day was associated with the greatest benefit (one RCT comprising 4731 patients; OR, 0.83 [95% CI, 0.70 to 0.99]; SUCRA, 89%; ARD, − 1.7% [95% CI, − 3.1 to − 0.1%]).

Five RCTs, comprising 6910 patients, addressed all-cause mortality outcome comparing a statin versus placebo/no statin. Low-quality evidence indicated that 7.9% of patients taking a statin for up to 5 years died compared with 7.7% patients taking placebo/no statin (OR, 1.04 [95% CI, 0.87 to 1.25]; ARD, 0.3% [95% CI, − 0.9 to 1.7%]). Uncertain estimates based on low- to very low-quality evidence prevented us from determining a reliable treatment hierarchy among different statins.

Eight RCTs, comprising 10,394 patients, addressed the ischemic stroke outcome comparing a statin versus placebo/no statin. High-quality evidence indicated that 7.6% of patients taking a statin experienced an ischemic stroke compared with 9.3% of patients taking placebo/no statin (OR, 0.81 [95% CI, 0.70 to 0.93]; ARD, − 1.6% [95% CI, − 2.6 to − 0.6%]). By comparing different statins, moderate- to high-quality evidence indicated that atorvastatin 80 mg/day (one RCT comprising 4731 patients; OR, 0.78 [95% CI, 0.64 to 0.94]; SUCRA, 68%; ARD, − 1.9 [95% CI, − 3.1 to − 0.5%]) and simvastatin 40 mg/day (four RCT comprising 3747 patients; OR, 0.83 [95% CI, 0.64 to 1.07]; SUCRA, 55%; ARD, − 1.5% [95% CI, − 3.1 to 0.6%]) were associated with the greatest benefits.

One RCT, comprising 4731 patients, addressed the ischemic stroke or TIA outcome comparing atorvastatin 80 mg/day versus placebo. High-quality evidence indicated that 15.9% of patients taking atorvastatin 80 mg/day experienced an ischemic stroke or TIA compared with 20.1% of patients taking placebo (OR, 0.75 [95% CI, 0.64 to 0.87]; ARD, − 4.2% [95% CI, − 6.2 to − 2.1%]).

Six RCTs, comprising 9976 patients, addressed the hemorrhagic stroke outcome comparing a statin versus placebo/no statin. Evidence indicated that 1.8% of patients taking a statin experienced a hemorrhagic stroke compared with 1.2% of patients taking placebo/no statin (OR, 1.54 [95% CI, 1.10 to 2.15]; ARD, 0.6% [95% CI, 0.1 to 1.3%]). A post hoc influence (leave-one-out) analysis showed that results were influenced by the largest trial. The meta-analysis of hemorrhagic strokes was sensitive to the exclusion of the Stroke Prevention by Aggressive Reduction in Cholesterol Levels (SPARCL) trial, and when excluded, the overall estimate moved towards no difference (OR, 1.35 [95% CI, 0.78 to 2.33]). We downgraded the evidence to low due to inconsistency and imprecision in the absolute risks for hemorrhagic stroke. Thus, the evidence suggests that the true role of statins in causing hemorrhagic stroke could be different from that observed in our overall estimate. The same caveat applies to single-agent analyses. Low-quality evidence indicated that atorvastatin 80 mg/day (one RCT comprising 4731 patients; OR, 1.68 [95% CI, 1.09 to 2.60]; SUCRA, 21%; ARD, 0.8% [95% CI, 0.1% to 1.8%]) and simvastatin 40 mg/day (four RCT comprising 3667 patients; OR, 1.69 [95% CI, 0.85–3.34]; SUCRA, 24%; ARD, 0.8% [95% CI, 0.2% to 2.6%]) were associated with potential harms.

Six RCTs, comprising 10,192 patients, addressed the cardiovascular event outcome comparing a statin versus placebo/no statin. High-quality evidence indicated that 22.8% patients taking a statin experienced a cardiovascular event compared with 28.0% patients taking placebo/no statin (OR, 0.75 [95% CI, 0.69 to 0.83]; ARD, − 5.4% [95% CI, − 6.8 to − 3.6%]). By comparing different statins, moderate- to high-quality evidence indicated that atorvastatin 80 mg/day (one RCT comprising 4731 patients; OR, 0.71 [95% CI, 0.62 to 0.80]; SUCRA, 91%; ARD, − 6.4% [95% CI, − 8.6 to − 4.3%]), simvastatin 40 mg/day (three RCT comprising 3569 patients; OR, 0.77 [95% CI, 0.67 to 0.90]; SUCRA, 70%; ARD, − 5.0% [95% CI, − 7.3 to − 2.1%]), and pravastatin 10 mg/day (one RCT comprising 1578 patients; OR, 0.82 [95% CI, 0.66 to 1.02]; SUCRA, 58%; ARD, − 3.8% [95% CI, − 7.6 to 4.0%]) were associated with the greatest benefits.

Three RCTs, comprising 6610 patients, addressed rhabdomyolysis, myalgia, or a rise in CK as a safety outcome comparing a statin versus placebo/no statin. Low-quality evidence indicated that 4.6% of patients taking a statin experienced rhabdomyolysis, myalgia, or a rise in CK compared with 4.8% of patients taking a placebo/no statin (OR, 0.95 [95% CI, 0.75 to 1.19]; ARD, − 0.2% [95% CI, − 1.2 to 0.9%]). Uncertain estimates based on low- to very low-quality evidence did not allow us to determine a reliable treatment hierarchy among different statins.

Heterogeneity estimates for standard pairwise meta-analysis and network meta-analysis in each outcome were low (Additional file 1: Tables S1-S10).

For each outcome, subgroup analyses by stroke subtypes at inclusion (i.e., studies including both cardioembolic and non-cardioembolic ischemic strokes versus those including non-cardioembolic ischemic strokes only), treatment dose (i.e., studies investigating statins at low- versus medium- versus high-intensity dose), and time from the first ischemic event to randomization (i.e., studies on patients randomized within 7 days versus beyond 1 month from the first ischemic event), as well as a sensitivity analysis including only trials classified as having a low risk of bias, did not show any significant difference compared to the overall analysis (Additional file 1: Tables S7-S10).

Based on a relative risk reduction or increase of 10%, a trial sequential analysis provided the required information size of 23,562 for all strokes (information deficit 13,164), 35,902 for all-cause mortality (information deficit 28,992), 29,238 for ischemic stroke (information deficit 18,844), and 65,217 for rhabdomyolysis, myalgia, or rise in CK (information deficit 58,607). The corresponding cumulative *Z* curves did not cross the trial sequential monitoring boundaries for benefit, harm, or futility. We cannot exclude, therefore, the risks of random type II error since the total sample sizes in the meta-analyses of these outcomes (from 6610 to 10,398) were considerably underpowered to identify a difference. The required information size was not quantifiable for hemorrhagic stroke due to the large distance between the accrued information and the required information. Conversely, a trial sequential analysis on the cardiovascular event outcome showed that the total sample size of 10,192 was higher than the required information size of 7819 calculated (information excess 2373); the corresponding cumulative *Z* curve crossed the trial sequential monitoring boundary for benefit after the first trial and never regressed. Accordingly, we can exclude random error as a cause of the finding for this outcome. Based on a relative risk reduction or increase of 20% (analyses conducted for secondary outcomes only), the trial sequential analysis computed a required information size of 6960 for ischemic stroke (information excess 3434), 70,999 for hemorrhagic stroke (information deficit 61,023), and 17,038 for the rhabdomyolysis, myalgia, or rise in CK outcome (information deficit 10,428). The corresponding cumulative *Z* curves for hemorrhagic stroke and the rhabdomyolysis, myalgia, or rise in CK outcome did not cross the trial sequential monitoring boundaries, while the cumulative *Z* curve for ischemic stroke outcome crossed the trial sequential monitoring boundary for benefit after the first trial and never regressed, together with a total sample size of 10,394 (i.e., higher than the required information size of 6960). The required information size was not quantifiable for cardiovascular event as the first information fraction exceeded 100% of the required information size boundary.

## Discussion

In this systematic review, network meta-analysis, and trial sequential analysis, we found that statins for secondary prevention in patients with ischemic stroke or TIA do not seem to modify all stroke and all cause-mortality outcomes; however, they reduce the relative risk of recurrent ischemic strokes by almost 20%, which corresponds to an absolute risk reduction of 1.6%, and the risk of cardiovascular events by more than 20%, which corresponds to an absolute risk reduction of 5.4%. These effects should be considered as clinically relevant based on our predefined minimal relevant differences and, by GRADE, are considered to be of high quality. Data from RCTs were not sufficient to assess if these effects are larger among patients at high risk, although guidelines in secondary prevention recommend treatment with statins particularly in patients with high LDL cholesterol levels to achieve a reduction of at least 50% through a high-intensity dose [6], based primarily on data from a secondary analysis of the SPARCL trial [40]. Furthermore, our meta-analysis shows a consistent effect within different groups of patients at risk by subtype of stroke, treatment dose, and time from first ischemic event to randomization. Our results suggest that the use of statins is safe: the most conservative analysis shows that statins might be associated with a higher relative risk of hemorrhagic stroke of about 50%, although only a minority of patients (less than 2%) would be exposed to the incremented risk corresponding to an absolute risk increase of 0.6%. The observed increased risk of hemorrhagic stroke is principally due to data from the SPARCL trial [33] in which the integrity of the intervention (statins and co-treatments, such as hypertensive therapies) was possibly not maintained over time. Thus, this finding could be an artifact. Results on all strokes, all-cause mortality, and the rhabdomyolysis, myalgia, or rise in CK outcome did not show relevant differences in risk between treated patients and controls, although the corresponding data were underpowered as shown by the trial sequential analysis. Finally, we found no differences among different statins. If any, the difference seems to be related to very high doses as atorvastatin 80 mg/day and simvastatin 40 mg/day were tested in a few trials.

While the effectiveness of statin therapy in preventing cardiovascular events in patients with prior ischemic stroke or TIA has been largely uncontested, recent investigations have raised questions as to the accuracy of data comparing the relative efficacy and safety of different statin drugs. Bero and colleagues pointed out that RCTs of head-to-head comparisons of statins with other drugs are more likely to report results and conclusions in favor of the product made by the sponsor versus the comparator drug [41]. The favoritism in study outcomes may be the consequence of non-equivalent doses or coding and analysis of outcomes [42]. While nearly 15 different statin drugs have been developed by pharmaceutical companies over the years, only a few drugs have dominated the market, supported by the results of various industry-sponsored trials. A 1996 study showing atorvastatin to most dramatically reduce LDL levels compared with its competitors simvastatin, lovastatin, fluvastatin, and pravastatin brought atorvastatin to the limelight, causing it to become one of the highest revenue-generating drugs of all time. Interestingly, the release of the drug in 1997 coincided with the US Food and Drug Administration’s approval of Direct-to-Consumer Pharmaceutical Advertising, which allowed for the broadcast advertisement of prescription drugs. These events made atorvastatin the main drug of choice, even though there is no strong evidence supporting large differences among statins in human or animal studies [43, 44]. The results of our network meta-analysis and trial sequential analysis caution the preferential use of a particular statin based on industry-sponsored studies, as different statin drugs appear to be comparable in the secondary prevention of all strokes, ischemic strokes, and all-cause mortality.

Even if the clinical effects of statins are similar, they can differ in other important dimensions such as pharmacokinetic properties. Statins show different susceptibilities to metabolism by different isoenzymes in the cytochrome CYP450 family. Drugs or food that inhibit CYP3A4 (e.g., macrolides, grapefruit) can markedly increase the plasma levels of simvastatin and lovastatin given their low bioavailability, and, to a less extent, that of atorvastatin, with a consequent increase in risk of rhabdomyolysis and muscular toxicity [45, 46]. Conversely, rosuvastatin and pravastatin do not undergo substantial metabolism via the CYP450 pathway [47].

The estimates of treatment effect from our study parallel that of previous reviews featuring standard pairwise meta-analyses on the same matter [13, 14, 48, 49], but are more precise due to our larger quantity of data and resulting statistical power. Accordingly, our findings, based on about 2200 additional patients, confirm those from a Cochrane review on interventions in the management of serum lipids for preventing stroke recurrence published in 2009, which reported a borderline statistical difference favoring the statin group compared with the placebo group in stroke recurrence [13]. Our results are also aligned with those reported in a systematic review on secondary prevention of non-cardioembolic stroke published in 2009 [48]. Finally, we confirm the data on statin use in an acute phase of ischemic stroke and TIA provided by two previous reviews, which reported no statistically significant difference in the effect of statins possibly due to the limited number of studies and consequent lack of power [14, 49].

The findings from this systematic review may be limited by the fact that we did not search for unpublished trials. Moreover, analyses were restricted by the amount of data in the included studies, the absence of head-to-head trials, and the scarcity of data on patients at high risk for which little can be concluded about the benefits and harms of statins. We did not perform a formal cost-effectiveness analysis. Some of the statistical techniques, despite being sophisticated, did not change the actual paradigm of the clinical treatment of strokes with statins. Notwithstanding the above limitations, this review also features strengths, including the implementation of a trial sequential analysis methodology to control the risk of false-positive results in meta-analysis owing to sparse data and the repetitive analyses of data [25–28], which adds some certainty to previous findings. We developed and published a protocol before we embarked on the review itself, and we conducted extensive searches of relevant databases. Finally, our meta-analysis, conducted in a well-defined vulnerable population, makes an in-depth evaluation of the possible differences between statins, limiting the role of possible distortions related to funding or biases. Our study further assessed the outcomes for which evidence can be considered conclusive.

## Conclusions

The findings from this network meta-analysis represent the most comprehensive evidence base to date that can guide best practice strategies for statin treatment in adults with a previous stroke or TIA. Statins do not appear to be better than placebo at preventing all strokes and all-cause mortality; however, they appear to reduce the risk of recurrent ischemic strokes and other cardiovascular events in patients with a previous stroke or TIA, with a limited risk of adverse events. The possibility of potential effect differences between statins cannot be dismissed given the potential limitations of the methodology, the complexity of patient populations at risk of stroke recurrence, and the uncertainties that may arise from the choice of dose or treatment setting. We hope that these results will assist in shared decision-making between patients and their clinicians and will inform the procurement of medicines within national health care systems.

## Additional file

Additional file 1: Network meta-analysis estimates and common standard deviation heterogeneity of primary and secondary outcomes for each comparison: all strokes (**Table S1.**), all-cause mortality (**Table S2.**), ischemic stroke (**Table S3.**), hemorrhagic stroke (**Table S4.**), cardiovascular event (**Table S5.**), and rhabdomyolysis, myalgia, or rise in creatine kinase (CK) (**Table S6.**). Treatments are reported in alphabetic order. Comparisons should be read from left to right. The estimate, odds ratio (OR), and 95% confidence interval are located at the intersection of the column-defining treatment and the row-defining treatment. An OR value below one favors the column-defining treatment for lower triangle, and the row-defining treatment for upper triangle. Significant results are bolded. Pairwise meta-analysis estimates of any statin versus placebo/no statin for primary and secondary outcomes overall, and by stroke subtypes at inclusion (**Table S7.**), treatment dose (**Table S8.**), and time from first ischemic event to randomization (**Table S9.**). **Table S10.** Sensitivity analysis of any statin versus placebo/no statin for primary and secondary outcomes overall, and including only trials classified as having low risk of bias. Table S11 Dataset underlying the findings of the meta-analyses. (DOCX 53 kb)

## Abbreviations

ACC/AHA: American College of Cardiology/American Heart Association
ARD: Absolute risk difference
CI: Confidence interval
CK: Creatine kinase
GRADE: Grading of Recommendation, Assessment, Development and Evaluation
ICTRP: International Clinical Trials Registry Platform
LDL: Low-density lipoprotein
NA: Not available
NIHSS: National Institutes of Health Stroke Scale
OR: Odds ratio
PRISMA: Preferred Reporting Items for Systematic Review and Meta-analysis
RCT: Randomized controlled trial
SPARCL: Stroke Prevention by Aggressive Reduction in Cholesterol Levels
SUCRA: Surface under the cumulative ranking curve area
TIA: Transient ischemic attack
TSA: Trial sequential analysis
