# Supplementary material for: Comparison of statins for secondary prevention in patients with ischemic stroke or transient ischemic attack: a systematic review and network meta-analysis
Source: BMC Med. 2019 Mar 26;17:67. doi: 10.1186/s12916-019-1298-5 (PMC6436237; doi:10.1186/s12916-019-1298-5)
Supplement: Supplementary file 1 — Network meta-analysis estimates and common standard deviation heterogeneity of primary and secondary outcomes for each comparison: all strokes (Table S1.), all-cause mortality (Table S2.), ischemic stroke (Table S3.), hemorrhagic stroke (Table S4.), cardiovascular event (Table S5.), and rhabdomyolysis, myalgia, or rise in creatine kinase (CK) (Table S6.). Treatments are reported in alphabetic order. Comparisons should be read from left to right. The estimate, odds ratio (OR), and 95% confidence interval are located at the intersection of the column-defining treatment and the row-defining treatment. An OR value below one favors the column-defining treatment for lower triangle, and the row-defining treatment for upper triangle. Significant results are bolded. Pairwise meta-analysis estimates of any statin versus placebo/no statin for primary and secondary outcomes overall, and by stroke subtypes at inclusion (Table S7.), treatment dose (Table S8.), and time from first ischemic event to randomization (Table S9.). Table S10. Sensitivity analysis of any statin versus placebo/no statin for primary and secondary outcomes overall, and including only trials classified as having low risk of bias. Table S11 Dataset underlying the findings of the meta-analyses. (DOCX 53 kb) [file 12916_2019_1298_MOESM1_ESM.docx]

**Additional file**

**Tables S1-S6** Network meta-analysis estimates and common standard deviation heterogeneity of primary and secondary outcomes for each comparison: all strokes (S1 Table), all-cause mortality (S2 Table), ischemic stroke (S3 Table), hemorrhagic stroke (S4 Table), cardiovascular event (S5 Table) and rhabdomyolysis, myalgia or rise in creatine kinase (CK) (S6 Table). Treatments are reported in alphabetic order. Comparisons should be read from left to right. The estimate, odds ratio (OR), and 95% confidence interval, is located at the intersection of the column-defining treatment and the row-defining treatment. An OR value below one favors the column-defining treatment for lower triangle, and the row-defining treatment for upper triangle. Significant results are in bold.

**Table S1** All strokes.

| Atorvastatin | 1.15 (0.81-1.64) | 1.19 (0.90-1.57) | **1.20 (1.01-1.43)** |
| --- | --- | --- | --- |
| 0.87 (0.61-1.24) | Pravastatin | 1.03 (0.71-1.50) | 1.04 (0.76-1.42) |
| 0.84 (0.64-1.11) | 0.97 (0.67-1.41) | Simvastatin | 1.01 (0.82-1.25) |
| **0.83 (0.70-0.99)** | 0.96 (0.71-1.31) | 0.99 (0.80-1.23) | Placebo/No Statin |

Common standard deviation heterogeneity: 2.063e-14

**Table S2** All-cause mortality.

| Atorvastatin | 1.20 (0.72-1.99) | 1.00 (0.06-16.24) | 0.85 (0.40-1.80) | 0.97 (0.80-1.19) |
| --- | --- | --- | --- | --- |
| 0.83 (0.50-1.38) | Pravastatin | 0.83 (0.05-13.97) | 0.70 (0.30-1.68) | 0.81 (0.51-1.29) |
| 1.00 (0.06-16.25) | 1.20 (0.07-20.11) | Rosuvastatin | 0.85 (0.05-14.99) | 0.97 (0.06-15.72) |
| 1.18 (0.55-2.52) | 1.42 (0.60-3.37) | 1.18 (0.07-20.97) | Simvastatin | 1.15 (0.55-2.39) |
| 1.03 (0.84-1.25) | 1.23 (0.77-1.96) | 1.03 (0.06-16.55) | 0.87 (0.42-1.80) | Placebo/No Statin |

Common standard deviation heterogeneity: 7.122e-13

**Table S3** Ischemic stroke.

| Atorvastatin | 1.19 (0.79-1.79) | 0.51 (0.07-4.02) | 1.07 (0.78-1.46) | **1.29 (1.07-1.56)** |
| --- | --- | --- | --- | --- |
| 0.84 (0.56-1.26) | Pravastatin | 0.43 (0.05-3.45) | 0.89 (0.57-1.39) | 1.08 (0.75-1.55) |
| 1.95 (0.25-15.28) | 2.33 (0.29-18.64) | Rosuvastatin | 2.08 (0.26-16.41) | 2.52 (0.32-19.55) |
| 0.94 (0.68-1.29) | 1.12 (0.72-1.74) | 0.48 (0.06-3.80) | Simvastatin | 1.21 (0.94-1.56) |
| **0.78 (0.64-0.94)** | 0.92 (0.64-1.33) | 0.39 (0.05-3.09) | 0.83 (0.64-1.07) | Placebo/No Statin |

Common standard deviation heterogeneity: 4.404e-10

**Table S4** Hemorrhagic stroke.

| Atorvastatin | 0.59 (0.24-1.47) | 1.00 (0.44-2.26) | **0.59 (0.38-0.92)** |
| --- | --- | --- | --- |
| 1.70 (0.68-4.25) | Pravastatin | 1.70 (0.59-4.91) | 1.01 (0.45-2.26) |
| 1.00 (0.44-2.25) | 0.59 (0.20-1.69) | Simvastatin | 0.59 (0.30-1.18) |
| **1.68 (1.09-2.60)** | 0.99 (0.44-2.22) | 1.69 (0.85-3.34) | Placebo/No Statin |

Common standard deviation heterogeneity: 1.198e-07

**Table S5** Cardiovascular event.

| Atorvastatin | 1.16 (0.90-1.50) | 2.00 (0.90-4.42) | 1.10 (0.90-1.34) | **1.42 (1.24-1.62)** |
| --- | --- | --- | --- | --- |
| 0.86 (0.67-1.12) | Pravastatin | 1.72 (0.76-3.89) | 0.95 (0.72-1.24) | 1.22 (0.98-1.53) |
| 0.50 (0.23-1.11) | 0.58 (0.26-1.31) | Rosuvastatin | 0.55 (0.25-1.22) | 0.71 (0.32-1.55) |
| 0.91 (0.75-1.11) | 1.06 (0.81-1.38) | 1.82 (0.82-4.04) | Simvastatin | **1.29 (1.11-1.50)** |
| **0.71 (0.62-0.80)** | 0.82 (0.66-1.02) | 1.41 (0.64-3.09) | **0.77 (0.67-0.90)** | Placebo/No Statin |

Common standard deviation heterogeneity: 7.542e-08

**Table S6** Rhabdomyolysis, myalgia or rise in creatine kinase.

| Atorvastatin | 1.97 (0.64-6.07) | 0.83 (0.18-3.83) | 1.08 (0.85-1.37) |
| --- | --- | --- | --- |
| 0.51 (0.16-1.56) | Pravastatin | 0.42 (0.06-2.72) | 0.55 (0.18-1.65) |
| 1.21 (0.26-5.58) | 2.38 (0.37-15.44) | Rosuvastatin | 1.31 (0.29-5.94) |
| 0.92 (0.73-1.17) | 1.82 (0.61-5.46) | 0.76 (0.17-3.47) | Placebo/No Statin |

Fixed-effect model used as only one study was available for each direct comparison.

**Tables S7-S9** Pairwise meta-analysis estimates of any statin versus placebo/no statin for primary and secondary outcomes overall, and by stroke subtypes at inclusion (Table S7), treatment dose (Table S8), and time from the first ischemic event to randomization (Table S9).

**Table S7** Subgroup analysis by stroke subtypes at inclusion.

| **Outcome** | **Overall OR (95% CI) [number of studies]^a^** | **OR (95% CI) by stroke subtypes [number of studies]**^b^ | | **Test for heterogeneity** |
| --- | --- | --- | --- | --- |
|  |  | *Both cardio and non-cardio* | *Non-cardio* |  |
| All strokes | 0.90 (0.80-1.02) [n=7] | 1.00 (0.81-1.23) [n=5] | 0.85 (0.73-1.00) [n=2] | p=0.23 |
| All-cause mortality | 1.04 (0.87-1.25) [n=5] | 0.87 (0.42-1.80) [n=2] | 1.06 (0.88-1.27) [n=3] | p=0.61 |
| Ischemic stroke | 0.81 (0.70-0.93) [n=8] | 0.83 (0.64-1.07) [n=4] | 0.80 (0.68-0.95) [n=4] | p=0.84 |
| Hemorrhagic stroke | 1.54 (1.10-2.15) [n=6] | 1.69 (0.85-3.34) [n=4] | 1.44 (0.90-2.31) [n=2] | p=0.76 |
| Cardiovascular event | 0.75 (0.69-0.83) [n=6] | 0.77 (0.67-0.90) [n=3] | 0.78 (0.64-0.96) [n=3] | p=0.67 |
| Rhabdomyolysis, myalgia or rise in CK | 0.95 (0.75-1.19) [n=3] | NA | 0.95 (0.75-1.19) [n=3] | NA |

^a^All I^2^=0%
^b^Cardio=cardioembolic ischemic stroke; Non-Cardio=non-cardioembolic ischemic stroke.

**Table S8** Subgroup analysis by treatment dose.

| **Outcome** | **Overall OR (95% CI) [number of studies]**^a^ | **OR (95% CI) by treatment dose [number of studies]** | | | **Test for heterogeneity** |
| --- | --- | --- | --- | --- | --- |
|  |  | *Low-intensity dose* | *Moderate-intensity dose* | *High-intensity dose* |  |
| All strokes | 0.90 (0.80-1.02) [n=7] | 0.93 (0.67-1.30) [n=1] | 1.00 (0.81-1.23) [n=5] | 0.83 (0.70-0.99) [n=1] | p=0.41 |
| All-cause mortality | 1.04 (0.87-1.25) [n=5] | 1.23 (0.77-1.96) [n=1] | 0.87 (0.42-1.80) [n=2] | 1.03 (0.84-1.25) [n=2] | p=0.69 |
| Ischemic stroke | 0.81 (0.70-0.93) [n=8] | 0.92 (0.64-1.33) [n=1] | 0.83 (0.64-1.07) [n=5] | 0.66 (0.25-1.75) [n=2] | p=0.67 |
| Hemorrhagic stroke | 1.54 (1.10-2.15) [n=6] | 0.99 (0.44-2.22) [n=1] | 1.69 (0.85-3.34) [n=4] | 1.68 (1.09-2.60) [n=1] | p=0.50 |
| Cardiovascular event | 0.75 (0.69-0.83) [n=6] | 0.82 (0.66-1.02) [n=1] | 0.77 (0.67-0.90) [n=3] | 0.89 (0.47-1.69) [n=2] | p=0.56 |
| Rhabdomyolysis, myalgia or rise in CK | 0.95 (0.75-1.19) [n=3] | 1.82 (0.61-5.46) [n=1] | NA | 0.92 (0.73-1.16) [n=2] | p=0.23 |

^a^All I^2^=0%

**Table S9** Subgroup analysis by time from the first ischemic event to randomization.

| **Outcome** | **Overall estimates (95% CI) [number of studies]^a^** | **OR (95% CI) by time from first ischemic event to randomization [number of studies]** | | **Test for heterogeneity** |
| --- | --- | --- | --- | --- |
|  |  | *≤7 days* | *>1 month* |  |
| All strokes | 0.90 (0.80-1.02) [n=7] | 1.00 (0.49-2.02) [n=3] | 0.90 (0.79-1.02) [n=3] | p=0.55 |
| All-cause mortality | 1.04 (0.87-1.25) [n=5] | 0.88 (0.43-1.78) [n=3] | 1.06 (0.88-1.27) [n=2] | p=0.62 |
| Ischemic stroke | 0.81 (0.70-0.93) [n=8] | 0.92 (0.46-1.84) [n=5] | 0.80 (0.70-0.93) [n=3] | p=0.71 |
| Hemorrhagic stroke | 1.54 (1.10-2.15) [n=6] | 0.83 (0.12-5.70) [n=3] | 1.57 (1.12-2.20) [n=3] | p=0.52 |
| Cardiovascular event | 0.75 (0.69-0.83) [n=6] | 1.11 (0.62-1.98) [n=3] | 0.75 (0.68-0.82) [n=3] | p=0.19 |
| Rhabdomyolysis, myalgia or rise in CK | 0.95 (0.75-1.19) [n=3] | 0.76 (0.17-3.47) [n=1] | 1.04 (0.63-1.73) [n=2] | p=0.78 |

^a^All I^2^=0%

**Table S10** Sensitivity analysis of any statin versus placebo/no statin for primary and secondary outcomes overall, and including only trials classified as having low risk of bias.

| **Outcome** | **Overall OR (95% CI) [number of studies]^a^** | **OR (95% CI) including only trials classified as having low risk of bias [number of studies]** |
| --- | --- | --- |
| All strokes | 0.90 (0.80-1.02) [n=7] | 0.98 (0.81-1.17) [n=4] |
| All-cause mortality | 1.04 (0.87-1.25) [n=5] | 1.19 (0.76-1.85) [n=3] |
| Ischemic stroke | 0.81 (0.70-0.93) [n=8] | 0.86 (0.69-1.06) [n=5] |
| Hemorrhagic stroke | 1.54 (1.10-2.15) [n=6] | 1.26 (0.63-2.52) [n=4] |
| Cardiovascular event | 0.75 (0.69-0.83) [n=6] | 0.80 (0.70-0.90) [n=4] |
| Rhabdomyolysis, myalgia or rise in CK | 0.95 (0.75-1.19) [n=3] | 1.35 (0.56-3.28) [n=2] |

^a^All I^2^=0%

**Table S11** Dataset underlying the findings of the meta-analyses.

| **Study** | **Total randomized (intervention / control)** | **All strokes (intervention / control)** | **All-cause mortality (intervention / control)** | **Ischemic stroke (intervention / control)** | **Ischemic stroke or TIA (intervention / control)** | **Hemorrhagic stroke (intervention / control)** | **Cardiovascular event (intervention / control)** | **Rhabdomyolysis, myalgia or rise in CK (intervention / control)** |
| --- | --- | --- | --- | --- | --- | --- | --- | --- |
| White 2000 | 156/176 | 13/13 | NA | NA | NA | NA | NA | NA |
| Collins 2004 | 1,643/1,637 | 169/170 | NA | 100/122 | NA | 21/11 | 406/488 | NA |
| Amarenco 2006 | 2,365/2,366 | 265/311 | 216/211 | 218/274 | 375/476 | 55/33 | 530/687 | 140/151 |
| Kennedy 2007 | 99/95 | 12/9 | NA | 12/9 | NA | 0/0 | NA | NA |
| Yakusevich 2012 | 86/97 | 5/7 | 13/16 | 4/7 | NA | 1/0 | 10/12 | NA |
| Hosomi 2015 | 793/785 | 74/78 | 42/34 | 62/66 | NA | 12/12 | 199/228 | 9/5^a^ |
| Ueno 2015 | 12/12 | NA | NA | 1/1 | NA | NA | NA | NA |
| Heo 2016 | 155/159 | NA | 1/1 | 0/3 | NA | NA | 16/12 | 3/4 |
| Montaner 2016 | 50/54 | 0/2 | 2/3 | 0/0 | NA | 0/2 | 0/2 | NA |

^a^Denominators for rhabdomyolysis, myalgia or rise in CK were “patients who received at least one dose of study drug in the pravastatin group and all patients who were assigned to the control group” as reported by the authors (i.e., 780/785).
